# Avoidable costs of physical treatments for chronic back, neck and shoulder pain within the Spanish National Health Service: a cross-sectional study

**DOI:** 10.1186/1471-2474-12-287

**Published:** 2011-12-21

**Authors:** Pedro Serrano-Aguilar, Francisco M Kovacs, Jose M Cabrera-Hernández, Juan M Ramos-Goñi, Lidia García-Pérez

**Affiliations:** 1 Health Technology Assessment Unit. Canary Islands Health Service. Government of the Canary Islands, Santa Cruz de Tenerife, Spain; 2 CIBER Epidemiología y Salud Pública (CIBERESP), Barcelona, Spain; 3 Fundación Kovacs, Palma de Mallorca, Spain; 4 Spanish Back Pain Research Network (REIDE), Palma de Mallorca, Spain; 5 General Directorate of Health Care Programmes. Canary Islands Health Service. Government of the Canary Islands, Santa Cruz de Tenerife, Spain; 6 Fundación Canaria de Investigación y Salud (FUNCIS), Santa Cruz de Tenerife, Spain; 7 Servicio de Evaluación, Dirección del Servicio Canario de la Salud, Pérez de Rozas 5, 4th floor, 38004 Santa Cruz de Tenerife, Canary Islands, Spain

**Keywords:** Physical therapy, Treatment costs, Evidence based practice, Avoidable costs, Back pain, Neck pain, Shoulder pain

## Abstract

**Background:**

Back, neck and shoulder pain are the most common causes of occupational disability. They reduce health-related quality of life and have a significant economic impact. Many different forms of physical treatment are routinely used. The objective of this study was to estimate the cost of physical treatments which, despite the absence of evidence supporting their effectiveness, were used between 2004 and 2007 for chronic and non-specific neck pain (NP), back pain (BP) and shoulder pain (SP), within the Spanish National Health Service in the Canary Islands (SNHSCI).

**Methods:**

Chronic patients referred from the SNHSCI to private physical therapy centres for NP, BP or SP, between 2004 and 2007, were identified. The cost of providing physical therapies to these patients was estimated. Systematic reviews (SRs) and clinical practice guidelines (CPGs) for NP, BP and SP available in the same period were searched for and rated according to the Oxman and AGREE criteria, respectively. Those rated positively for ≥70% of the criteria, were used to categorise physical therapies as Effective; Ineffective; Inconclusive; and Insufficiently Assessed. The main outcome was the cost of physical therapies included in each of these categories.

**Results:**

8,308 chronic cases of NP, 4,693 of BP and 5,035 of SP, were included in this study. Among prescribed treatments, 39.88% were considered Effective (physical exercise and manual therapy with mobilization); 23.06% Ineffective; 13.38% Inconclusive, and 23.66% Insufficiently Assessed. The total cost of treatments was € 5,107,720. Effective therapies accounted for € 2,069,932.

**Conclusions:**

Sixty percent of the resources allocated by the SNHSCI to fund physical treatment for NP, BP and SP in private practices are spent on forms of treatment proven to be ineffective, or for which there is no evidence of effectiveness.

## Background

Back, neck and shoulder problems are the most common causes of pain and occupational disability [1-3]. They reduce health-related quality of life (HRQL) and have a significant economic impact [4-8]. These musculoskeletal disorders are usually non-specific, which means that pain cannot be attributed to any specific structural cause and is believed to originate from soft tissues [9]. Their lifetime prevalence is 50-70% and they are among the most common reasons for primary care visits in Spain [10-14].

The Spanish National Health Service is a public health insurance system with universal coverage which provides free health care to every resident in Spain. Within the Spanish National Health Service (SNHS), primary care practices and hospitals are owned and managed by the government. Private hospitals and private primary care practices which are owned and managed by private entities exist in parallel to the SNHS, and operate independently. In 2008, 72.5% of total health care expenditure in Spain came from governmental funding [15]. The SNHS can choose to refer patients to private practices in order to shorten waiting times, usually for non-urgent, non life-threatening conditions, such as physical therapy for musculoskeletal complaints, or surgery for cataract or abdominal hernia. In such cases, the SNHS fully covers the cost of procedures performed by the private practices on the patients it refers. The SNHS is managed at the regional level, and fully financed by national taxes, although some regional governments add local taxes to provide additional funding. The Spanish National Health Service in the Canary Islands (SNHSCI) covers a population of two million individuals, and outsources approximately 70% of physical treatments to the private sector. Physicians at the SNHSCI make the diagnosis and treatment recommendations, but clinicians employed by the private practices have the ability to adjust or change treatments according to each patient's clinical response. The clinical management of shoulder, neck or back pain is not regulated by compulsory clinical guidelines, and clinicians are free to choose whether or not to follow the European and Spanish evidence-based clinical guidelines for low back pain [16,17].

Many different forms of physical treatment are routinely provided within the SNHS. The objectives of this study were to: a) identify the forms of physical therapies used for treating chronic non-specific neck (NP), back (BP) and shoulder pain (SP) between 2004 and 2007, within routine practice in the SNHSCI; b) classify these forms of treatments according to evidence on their effectiveness available at the time; and c) estimate the cost of physical treatments which had either been proven ineffective or not shown to be effective.

## Methods

### Identification of the forms of physical treatments used

The Information System for private Hospital contracts (ISHC), is a governmental database which includes data from patients in the SNHSCI, who were referred to private practices. In the case of patients with musculoskeletal problems who are referred for physical therapy, the database gathers demographic data, patient's diagnosis (ICD-9-CM) and the type and number of physical therapies received.

In Spain, occupational diseases and work-related injuries are not managed by the National Health Service, but by separate workers' compensation institutions. As a result, occupational diseases and work-related injuries are not included in the ISHC.

The type and number of physical treatments funded by the SNHSCI from January 1st, 2004 to December 31st, 2007, for patients over 18 years for non-specific chronic NP, BP and SP (ICD-9-CM-723.1, ICD-9-CM-724.2 and ICD-9-CM-726.1) were identified using the ISHC database. Chronic NP, BP or SP was defined as an episode lasting 12 or more weeks [18]. Only patients who initiated treatment at least 12 weeks after seeking care for SP, NP or BP (i.e., chronic cases) were included in the study. Treatments for other conditions were not considered.

The unit of analysis was every case of non-specific chronic NP, BP and SP identified in the ISHC database, irrespectively of whether a single patient received treatment on several occasions for the same or different conditions during the study period.

### Evidence on the effectiveness of the different forms of physical treatment

An electronic search of clinical practice guidelines (CPGs) and systematic reviews (SRs) on NP, BP and SP published before 31st of December, 2007, was carried out in the following databases: MEDLINE, EMBASE, The Centre for Reviews and Dissemination (University of York), Cochrane Library Plus, Trip Database, Pubgle, The National Guideline Clearinghouse, Fisterra, Guiasalud, The Web of the Back (Kovacs Foundation), The European Commission Research Directorate General (University of Bergen, Norway) and the Institute for Clinical Systems Improvement (Minnesota, USA) [See additional file 1: Search strategy].

CPGs and SRs focusing on chronic nonspecific NP, BP and SP, and covering any form of physical treatment, were selected. Their quality was assessed independently by two assessors, and disagreements between them were discussed and resolved by the first author. Quality assessment was based on the AGREE instrument for CPGs and the Oxman scale criteria for SRs. The AGREE instrument consists of 23 key items organised in six domains, to capture separate dimensions of CPG quality [19]. These domains are: scope and purpose, stakeholder involvement, rigour of development, clarity and presentation, applicability, and editorial independence. The Oxman scale has been widely used to assess the quality and reliability of SR. The scale contains ten items on the internal and external validity of SRs (search methods, criteria to include studies and avoiding bias, criteria to assess validity, methods of combination of study results, quality of reporting and data supporting conclusions) [20]. Only CPGs and SRs which were positively rated in ≥ 70% of the dimensions explored by these instruments were considered of "high quality" and were included in this study.

According to the conclusions of the SRs and CPGs included, physical treatments were classified into four categories [21]: Effective; of Inconclusive Effectiveness; Insufficiently assessed and Ineffective. Each form of treatment was classified in the best category in which any of the included CPGs or SRs had ranked it. For instance, it was sufficient for a therapy to be considered effective in one of the CPGs or SRs included, to be classified as such in this study. According to this conservative assumption any form of treatment labelled as "exercise" was considered to be effective.

### Cost estimation

The fees paid by the SNHSCI to private practices cover 35 sessions of physical therapy for SP patients, and 30 for NP and BP. These fees remain constant irrespectively of the type of physical treatment actually provided during each session. In fact, the physicians in private practices can modify the physical treatment dispensed, depending on patient's clinical evolution and their own clinical criteria. In practice, most patients included in this study actually received several forms of physical treatment (Table 1). Since the SNHSCI pays for a "package" of 30-35 sessions, and the type of physical therapies actually performed during these sessions varies from one patient to the other, it was impossible to estimate the amount paid by the SNHS for each particular form of physical treatment.

**Table 1:** Costs of each form of therapy used for treating non-specific chronic neck pain (NP), back pain (BP) and shoulder pain (SP).

		NP: 8308 cases			BP: 4693 cases			SP: 5035 cases			Total: 18036 cases	
	**Unit cost^a ^(€)** **(U)**	**N° of Procedures** **(P)**	**N° of Sessions** **(S)**	**Costs (€)** **(UxPxS)**	**N° of Procedures** **(P)**	**N° of Sessions** **(S)**	**Costs (€)** **(UxPxS)**	**N° of Procedures** **(P)**	**N° of Sessions** **(S)**	**Costs (€)** **(UxPxS)**	**Total N° of Procedures**	**Total Costs (€)**

**Exercises**	1.25	10440^b^	30	391509	6613^b^	30	248006	7194^b^	35	314729	18036	954243

**Manual mobilization**	4.5	7366	30	994379	3500	30	472489	3056	35	481346	13922	1948214

**Thermotherapy**	2	4392	30	263544	2807	30	168405	1591	35	111394	8791	543344

**TENS**	1	3885	30	116563	2006	30	60180	2435	35	85219	8326	261963

**Ultrasounds**	1.25	3386	30	126972	1686	30	63209	2665	35	116574	7736	306754

**Hot compresses**	1.25	1983	30	74371	1123	30	42130	944	35	41318	4051	157819

**Electrical stimulation**	2.5	1857	30	139265	1453	30	108975	1170	35	102340	4479	350579

**Traction**	1	1230	30	36907	281	30	8432	249	35	8699	1760	54037

**Short wave**	2.5	948	30	71082	418	30	31363	1594	35	139506	2960	241951

**Cutaneous laser**	3	311	30	27964	120	30	10772	688	35	72198	1118	110935

**Iontophoresis**	2.5	216	30	16223	152	30	11420	740	35	64778	1109	92421

**Magnetotherapy**	3	198	30	17862	270	30	24340	412	35	43255	881	85458

**Total**		36213		2276642	20430		1249722	22737		1581356	79381	5107720

National Health Service in the Canary Islands (ISCH), 2004 to 2007

^a^*Unit costs *1 session for 1 procedure or case

^b^No. of procedures for exercises are higher than the number of cases for NP, BP and SP due to different types of specific exercises prescribed, with additional costs, for the same case.

Therefore, the costs were estimated using data provided by the private subcontractor to which SP, NP and BP patients were referred from the SNHSCI. These data only included personnel and equipment costs incurred by the private practices in order to provide each form of physical treatment to SP, NP and BP patients referred from the SNHSCI (Table 1). For treatments which are provided to several patients simultaneously (e.g., thermotherapy or some forms of exercise), the conservative assumption that each group comprised two patients, was made. Therefore, for such treatments, unit cost per patient was estimated by dividing the cost of treatment by two.

Total cost for each therapy was obtained by multiplying the unit cost by number of sessions and by number of patients (Table 1). For example, the costs associated with exercises for NP patients were obtained as follows: unit cost per patient per session (€1.25) × number of sessions per patient (30) × number of different types of exercises applied (10,440) in all included cases.

Avoidable costs were defined as costs of treatments which had been classified as "Ineffective", "of Inconclusive Effectiveness" or "Insufficiently Assessed". All costs are expressed in inflation-adjusted, 2008 Euros (€).

## Results

From 2004 to 2007, the SNHSCI covered the cost of physical treatment administered in private practices for 8,308 cases of chronic nonspecific NP, 4,693 of BP and 5,035 of SP. In 1,082 (5.99%) out of these 18,036 cases were simultaneously suffering from other musculoskeletal disorders.

The average age of the patients was 53.85 years (SD: ±14.52) and 73.25% were women. On average, patients treated for NP, BP and SP received 4.36, 4.35 and 4.52 different forms of physical therapies respectively. The forms of treatment most commonly administered, its frequencies and number of sessions are shown in Table 1.

The SR search detected 806 references, corresponding to 525 original studies. Sixty-three of them were relevant to this study, and 19 rated positively in ≥70% of the Oxman criteria [22-40]. The CPG search detected 385 references, corresponding to 234 individual guidelines. Thirty-four of them were relevant to this study, and 12 rated positively in ≥70% of the AGREE criteria [16,17,21,41-49].

According to the results and recommendations from these SRs and CPGs exercise is effective for NP [21,23,32,35,41,42,45], BP [16,17,28,31,37,46,47] and SP [31,32,35,38,44,48,49]. Some SRs an d CPGs also recommend adding manual therapy with mobilization for NP [34,43,45] and SP [31,48]. All the other forms of physical treatment are either ineffective, of inconclusive effectiveness, or have not been adequately assessed. Table 2 summarizes the evidence supporting the treatments for these conditions.

**Table 2:** Classification of physical therapies for neck, back and shoulder pain according to the evidence on effectiveness from selected systematic reviews (SRs) and clinical practice guidelines (CPGs)^a^

Form of treatment	Neck Pain							Back Pain							Shoulder Pain						
	**SRs**				**CPGs**		**C†**	**SRs**				**CPGs**		**C**	**SRs**				**CPGs**		**C**

	**E‡**	**IE§**	**IA|**	**I¶**	**R#**	NR*		**E**	**IE**	**IA**	**I**	**R**	**NR**		**E**	**IE**	**IA**	**I**	**R**	**NR**	

**Exercises**	^21, 30, 33^				^19, 39, 40, 45^		**E**	^26, 29, 35^				^42, 43, 46, 47^		**E**	^29, 30, 33, 36^				^44, 48, 49^		**E**

**Manual mobilization**	^32^	^34^			^41, 45^		**E**			^43^	^28^			**I**	^29^				^48^		**E**

**Massage**		^20^				^19^	**IE**	ND	ND	ND	ND	ND	ND	**IA**	ND	ND	ND	ND	ND	ND	**IA**

**Thermotherapy**	ND	ND	ND	ND	ND	ND	**IA**			^27, 43^			^42^	**IE**	ND	ND	ND	ND	ND	ND	**IA**

**TENS**		^22^					**IE**			^35, 43^	^24, 36^		^42^	**I**	ND	ND	ND	ND	ND	ND	**IA**

**Ultrasounds**						^19^	**IE**			^35, 43^			^42^	**IE**						^49^	**IE**

**Hot compresses**	ND	ND	ND	ND	ND	ND	**IA**			^43^			^42^	**IE**	ND	ND	ND	ND	ND	ND	**IA**

**Electrical stimulation**				^22^			**I**			^35, 43^			^42^	**IE**	ND	ND	ND	ND	ND	ND	**IA**

**Traction**						^19^	**IE**			^35, 43^	^25^		^42^	**I**	ND	ND	ND	ND	ND	ND	**IA**

**Short wave**	ND	ND	ND	ND	ND	ND	**IA**			^35, 43^			^42^	**IE**				^37, 38^			**I**

**Cutaneous laser**				^31^			**I**			^35, 43^	^23^		^42^	**I**						^49^	**IE**

**Iontophoresis**				^22^			**I**	ND	ND	ND	ND	ND	ND	**IA**	ND	ND	ND	ND	ND	ND	**IA**

**Magnetotherapy**				^22^			**I**	ND	ND	ND	ND	ND	ND	**IA**	ND	ND	ND	ND	ND	ND	**IA**

^a^SRs and CPGs are cited by its reference number.

†*C *Classification in this study. ‡*E *effective; §*IE *inconclusive effectiveness; || *IA *insufficiently assessed, ¶ *I *ineffective. #*R *CPGs recommending the use of the therapy. *NR CPGs not recommending the therapy. *ND *No data available from SRs or CPGs.

**Figure 1:**
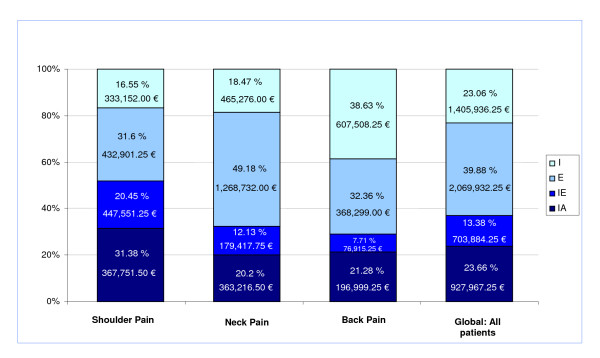
**Distribution of physical therapies administered according to the evidence of effectiveness and its corresponding costs to patients with chronic nonspecific NP, BP or SP in Spanish National Health Service**. Legend: I: Proven ineffective, E: Proven effective, IE: Inconclusive evidence on effectiveness, IA: Insufficiently Assessed.

In total, 79,381 treatments were applied; 39.88% were classified as effective, 23.06% as ineffective, and 13.38% as of inconclusive effectiveness. The clinical value of the remaining 23.66% had not been properly assessed (Figure 1).

Overall, 3,037,788 (59.5%) of the € 5,107,720 spent on physical treatment for these conditions between 2004 and 2007, was spent on technologies which have proven to be ineffective, or for which there is no evidence of effectiveness (Figure 1).

## Discussion

These results show that as much as 60% of the resources spent by the SNHSCI on physical treatments for non-specific chronic NP, BP and SP between 2004 and 2007, were allocated to treatments that had been found to be either ineffective or for which there was no evidence of effectiveness. This means that, in that period, over 3 million Euros from taxpayers' contributions was handed over to private centres which had been awarded contracts by the government to apply treatments which lacked any evidence of effectiveness, or which had shown to be ineffective.

This is a very conservative estimation of the total cost, as it does not factor in data on the costs deriving from physical treatments administered in public hospitals and primary care practices run by the SNHSCI; only data from patients who were referred to private practices were included, representing 70% of patients receiving physical treatment for these conditions. In addition, it was sufficient for a therapy to be considered effective in a single CPG or SR, to be categorised as such in this study. Any form of treatment labelled as "exercise" was considered to be effective, irrespective of the type of exercise, number of sessions, patients' compliance or whether it was administered appropriately or inappropriately. Exercise was considered to be effective irrespectively of its effect size, whereas previous studies suggest that improvements in pain or disability of less than 30% of baseline value are clinically irrelevant for patients with NP and BP [50-52]. Moreover, only the provider's personnel and equipment costs directly related to physical therapies used for SP, NP and BP were taken into account, disregarding other costs (e.g., other provider's costs-facilities, financial costs, etc.- and profit), societal costs (work absenteeism associated with receiving physical therapy sessions, etc.), costs incurred by patients (transport, loss of earnings, etc.) and other costs for other institutions (insurance companies dealing with work-related accidents, private healthcare, etc.).

All the SRs and CPGs reviewed conclude that the quality of most clinical trials on physical treatments was low. The main shortcomings were related to randomization process, comparison treatments, masking procedures, losses to follow-up and missing data. This is likely to have led to an overestimation of the effect of physical treatments considered to be effective [21].

Inconsistencies in the prescription of different forms of treatments for patients with the same condition, may be due to the preferences of the different types of clinicians involved (primary care, rheumatology, rehabilitation, etc.), and the ready availability of these techniques. However, there are no validated criteria for selecting patients for whom a particular physical treatment would be indicated, or to decide the number of sessions they would need. In fact, there is even no evidence supporting the effectiveness of most of these forms of treatment or the cost/effectiveness of any of them, alone or in combination. These facts suggest that there may be ample room for improving the efficiency of the management of these syndromes in the SNHS. Ineffective technologies expose patients to risks, delays, expectations and costs which are unjustified [53]. Spending public funds on such procedures is inappropriate, and even more so in a context in which health resources are limited.

A number of recommendations have been issued regarding the procedures that health authorities should follow in order to ensure that the health technologies they finance are safe, effective and efficient [54]. Implementing these recommendations is likely to improve the efficacy of treatments and reduce wastage of healthcare resources, hence increasing the efficiency of available resources. In the context of an economic crisis, it is necessary to apply these measures before considering whether healthcare spending needs to be increased. This may require communication skills to explain it to clinicians, patients and public at large [55].

The search for SRs and CPGs was restricted to the 2003-2007 periods. It is possible that studies published afterwards may have changed, or will change, the evidence supporting the use of procedures included in this study. However, this study focused on quantifying the costs of procedures used in routine practice, despite the lack of supporting evidence at the time of use. In this regard, even if the evidence were to change based on future research, the use of these technologies in the 2004-2007 periods would continue to be inappropriate.

The results of this study illustrate the rationale for recommending disinvestment in specific health technologies. Disinvestment refers to the processes of reducing or discontinuing utilization of selected procedures and treatments [56]. Proactive disinvestment requires prior analyses of inappropriate variation in clinical practice, development of valid CPGs designed to update clinicians' education and practice, adequate information for patients, in order to improve consumer behaviour, organisational support within the health services for implementing these changes, economic incentives for providers, and incentives for improving the efficiency of purchasing decisions, including the elimination of funding for technologies which are ineffective and/or unsafe. Clinicians, consumers and providers of potentially discarded technologies may feel threatened by disinvestment, while beneficiaries, such as taxpayers and patients themselves, may be less aware of the advantages of such decisions. As a result, incentives to perpetuate the status quo may override the call for change [57].

However, disinvestment in technologies which are not evidence-based, would make funds available for implementing technologies which are not currently available in the SNHSCI despite having proven effective and cost/effective for NP and BP within the SNHS, such as neuro-reflexotherapy [58-60], and for further expanding the use of technologies proven effective for the conditions reviewed in this study, such as exercise (Table 1, Figure 1). Funds made available through disinvestment could also be used for supporting further research on the effectiveness and cost/effectiveness of physical procedures, which is sparse and of low scientific validity (Table 2).

In fact, results from this study suggest that there is a need for high-quality studies assessing the effectiveness and cost/effectiveness of different forms of physical therapy, individually and in combination. These should be randomized controlled clinical trials, with homogenous and sufficiently large samples, in which validated instruments should be used to assess clinically meaningful variables, and in which randomization, patients' assessment and data analysis should be masked. These trials should analyze both the statistical significance and clinical relevance of results. Since data on comparative effectiveness of different procedures are difficult to interpret when neither has previously shown superiority versus "sham" or placebo, these studies should compare different forms of physical treatment to the appropriate "sham" procedures as well as to other interventions. Taking into account that some treatments formerly believed to be useful for patients with acute BP proved to actually be harmful, when appropriate, these studies should consider including groups without any intervention [61]. The assessment of the results should also be made from the patient's point of view [54,62,63].

This study has some strengths. It contributes to the existing literature on non-adherence to CPGs by describing its economic consequences. Analyzed data were obtained through an information system which gathers information directly from routine clinical practice, leading to results which are directly applicable. Moreover, it analyzes data retrospectively, avoiding potential changes in referral patterns and selection of treatments which may occur when clinicians feel observed.

This study also has certain limitations. The Canary Islands' ISHC was one of the first health information systems to be implemented within the Spanish National Health Service, and it does not gather data on the criteria behind the prescription of any particular form of treatment. Moreover, it does not record additional treatments which are applied once the prescribed ones have started to be administered; therefore, patients may have actually undergone more forms of treatment than those which were identified in this study. The coding procedure is likely to be accurate, since it determines payments, but it may be influenced by the experience of the coders and their interest in administrative and/or clinical details. However, this is the only systematic and validated mechanism for post-implementation surveillance of physical therapies within the Spanish National Health Service.

This study included patients who, in addition to suffering from NP, BP or SP, also presented other concomitant musculoskeletal disorders which might have required physical treatment. In order to reduce contamination, the forms of physical treatment which were not used for NP, BP or SP were excluded. Nevertheless, it could be argued that some of the physical therapies attributed to the treatment of NP, BP or SP might have actually been used to treat concomitant conditions, leading to inappropriate assumptions. However, such co-morbidity was only present in 6% of cases. Therefore, we believe that this potential inaccuracy is unlikely to modify the results significantly.

These results were obtained from a single Spanish region, so generalisation of these findings should be discussed. The Canary Islands has a population of approximately 2 million, and a similar demographic structure, populations' age distribution, life expectancy and infant mortality to the average values of Spain [64]. The National Health Service in all of the 17 Spanish regions offers universal coverage and free access to treatments consistently across regions and includes the same physical treatments for SP, NP and BP [65]. The ratios of healthcare resources (professionals, infrastructure and technological equipment) in the Canary Islands are in line with the average values in the rest of Spain [66]. Moreover, the structure of healthcare and referral process to physical therapies and rehabilitation services is fairly homogeneous across the country, with a high proportion of services provided by private outpatient clinics subcontracted by regional health authorities, and the clinical management of BP patients is consistent across regions [67]. All this suggests that generalisability of these results to the rest of the Spanish National Health Service should not be a major concern.

No studies quantifying the amount of resources potentially wasted on non evidence-based physical therapies for SP, NP and BP in other countries have been found. However, approximately 50%, 65% and 94% of physiotherapists in the UK, US and Canada, respectively, use procedures which were classified in this study as non-evidence based (Table 2) [68]. In addition, approximately 55% of primary care practitioners in the US, recommend those forms of treatment [69]. In fact, only in the US more than 200 treatments are offered only for BP, most of which are not evidence-based [70,71]. With this non-evidence based approach, the cost of health care provided to low back pain patients in the US increased by 65% (constant dollars) from 1997 to 2005, without generating any improvements in outcomes [72]. In contrast to this, in The Netherlands, yearly costs derived from low back pain represented 1.7% of the Gross Domestic Product in 1995, and were reduced to 0.9% in 2002 and to 0.6% in 2007. This decrease was achieved without noticing deterioration in outcomes, and is attributed to the progressive implementation of an evidence-based clinical management [8,73]. Although available data do not reveal the exact amount of potential savings which could be made in other countries, results from this study are likely to be generalisable to other industrialised countries, suggesting that disinvesting in not-evidence based physical therapies for SP, NP and BP, would reduce costs without worsening patients' evolution, thus substantially improving efficiency.

This study did not assess actual patient's outcomes, and it might be argued that even therapies classified as "ineffective" could have some effects which previous clinical trials failed to detect. However, this study did not have a control group, and it would be inappropriate to assume that potential patients' improvement in routine practice necessarily corresponds to benefits deriving from the treatment, as opposed to unspecific factors (such as natural history or placebo). In fact, this study was not a clinical trial designed to assess the effectiveness of each form of treatment. It was a study of clinical practice designed to quantify the resources allocated to physical treatments which are supported by the existing evidence, and those which are not. Therefore, it classified the evidence supporting the use of each of treatment, based on the evidence from existing systematic reviews and evidence-based clinical guidelines. To this purpose, it seems appropriate to assume that a potential effect which is small or uncommon enough to remain undetected in the available trials, is likely to be clinically irrelevant.

## Conclusions

The average physical treatment applied in private practice to patients with chronic non-specific neck, back and shoulder pain referred from the Spanish National Health Service in the Canary Islands, includes 4.5 forms of treatment applied in 30-35 sessions. Between 2004 and 2007, only 40% of the treatments applied had previously shown to be effective, namely exercise and mobilization for certain cases. A conservative estimate suggests that treatments lacking any evidence of effectiveness represented 60% of total expenditure and accounted for approximately 3 million Euros. Over one million Euros were spent for treatments which had previously been shown to be ineffective.

## Abbreviations

HRQL: Health Related Quality of Life; NP: Neck pain; BP: Back pain; SP: Shoulder pain; ISCH: Information System for Private Hospital Contracts; ICD-9-CM: International Classification of Diseases 9th Revision, Clinical Modification; CPGs: Clinical Practice Guidelines; SRs: Systematic Reviews.

## Competing interests

The authors have no conflicts of interest to report. No benefits in any form have been or will be received by the authors or their institutions from any party harbouring economic interests related directly or indirectly to the subject of this article. Funds for this study came from governmental and not-for-profit scientific institutions, and no funds were received from any for-profit institution or entities linked to the health industry. While this study was designed and conducted, José María Cabrera-Hernández was involved in planning, assessing and financing treatments performed by private practices on patients referred from the SNHSCI. However, he had no links-either financial or otherwise- with any private practice or contractor, or incentives to increase or reduce the amount of payments made by the SNHSCI.

## Authors' contributions

PSA was responsible of the conception, design and organization of the research project; coordinated the literature revision as well as the writing and revision of the manuscript and approval the final version to be published. FMK participated in the literature review and execution of the research project; coordinated the writing and revision of the manuscript and approval of the final version to be published. JMCH participated in the conception and design of the project; coordinated the data gathering and analysis, and approval of the final version of the manuscript to be published. JMRG was co-responsible of the execution of the research project and data analyses. He participated in the writing of the results section and approval of the final version of the manuscript to be published. LGP was co-responsible for the execution of the research project and data analyses. She participated in the writing of the results section and approval of the final version of the manuscript to be published.

## Pre-publication history

The pre-publication history for this paper can be accessed here:

http://www.biomedcentral.com/1471-2474/12/287/prepub

## Supplementary Material

Additional file 1 **Search Strategy of Systematic Reviews and Clinical Practice Guidelines on electronic databases**. Click here for file
