# Supplementary material for: Avoidable costs of physical treatments for chronic back, neck and shoulder pain within the Spanish National Health Service: a cross-sectional study
Source: BMC Musculoskelet Disord. 2011 Dec 21;12:287. doi: 10.1186/1471-2474-12-287 (PMC3297536; doi:10.1186/1471-2474-12-287)
Supplement: Additional file 1 — Search Strategy of Systematic Reviews and Clinical Practice Guidelines on electronic databases. [file 1471-2474-12-287-S1.DOC]

**Additional file**

Search Strategy of Systematic Reviews and Clinical Practice Guidelines on electronic databases

**MEDLINE (Ovid)**

1) exp Shoulder Pain/

2) exp Rotator Cuff/

3) exp Bursitis/

4) frozen shoulder.ti,ab.

5) rotator cuff disorder$.ti,ab.

6) shoulder pain.ti,ab.

7) adhesive capsulitis.ti,ab.

8) 1-7 OR

9) exp Neck Pain/

10) exp Whiplash Injuries/

11) neck pain.ti,ab.

12) mechanical neck disorder$.ti,ab.

13) cervical pain.ti,ab.

14) whiplash.ti,ab.

15) 9-14 OR

16) exp Low Back Pain/

17) low back pain.ti,ab.

18) lumbar pain.ti,ab.

19) lumbago.ti,ab.

20) lumbalgia.ti,ab.

21) backache.ti,ab.

22) 16-21 OR

23) physical therapy.ti,ab.

24) short wave.ti,ab.

25) (manipulation OR mobilization OR rehabilitation OR massage OR traction OR ultrasound OR laser OR electrotherapy OR exercise$ OR management OR treatment$ OR training).ti,ab.

26) 23-25 OR

27) 8 OR 15 OR 22

28) 26 AND 27

29) (systematic adj review$).tw.

30) (data adj synthesis).tw.

31) (published adj studies).ab.

32) (data adj extraction).ab.

33) meta-analysis/

34) meta-analysis.ti.

35) 29-34 OR

36) comment.pt.

37) letter.pt.

38) editorial.pt.

39) 36-38 OR

40) 35 NOT 39

41) animal/

42) human/

43) 41 NOT (41 AND 42)

44) 40 NOT 43

45) 28 AND 44

46) limit 45 to yr="2003 - 2007"

47) guideline.pt.

48) guideline$.ti.

49) 47 OR 48

50) 28 AND 49

51) limit 50 to yr="2003 - 2007"

**EMBASE**

1) exp Shoulder Pain/

2) exp Rotator Cuff/

3) exp BURSITIS/

4) frozen shoulder.ti,ab.

5) rotator cuff disorder$.ti,ab.

6) shoulder pain.ti,ab.

7) adhesive capsulitis.ti,ab.

8) 1-7 OR

9) exp Neck Pain/

10) exp WHIPLASH INJURY/

11) neck pain.ti,ab.

12) mechanical neck disorder$.ti,ab.

13) cervical pain.ti,ab.

14) whiplash.ti,ab.

15) 9-14 OR

16) exp Low Back Pain/

17) low back pain.ti,ab.

18) lumbar pain.ti,ab.

19) lumbago.ti,ab.

20) lumbalgia.ti,ab.

21) backache.ti,ab.

22) 16-21 OR

23) 8 OR 15 OR 22

24) physical therapy.ti,ab.

25) short wave.ti,ab.

26) (manipulation or mobilization or rehabilitation or massage or traction or ultrasound or laser or electrotherapy or exercise$ or management or treatment$ or training).ti,ab.

27) 24-26 OR

28) 23 AND 27

29) exp Meta Analysis/

30) ((meta adj analy$) or metaanalys$).tw.

31) (systematic adj (review$1 or overview$1)).tw.

32) 29-31 OR

33) cancerlit.ab.

34) cochrane.ab.

35) embase.ab.

36) (psychlit or psyclit).ab.

37) (psychinfo or psycinfo).ab.

38) (cinahl or cinhal).ab.

39) science citation index.ab.

40) bids.ab.

41) 33-40 OR

42) reference lists.ab.

43) bibliograph$.ab.

44) hand-search$.ab.

45) manual search$.ab.

46) relevant journals.ab.

47) 42-46 OR

48) data extraction.ab.

49) selection criteria.ab.

50) 48 or 49

51) review.pt.

52) 50 and 51

53) letter.pt.

54) editorial.pt.

55) animal/

56) human/

57) 55 not (55 and 56)

58) 53 or 54 or 57

59) 32 or 41 or 47 or 52

60) 59 not 58

61) 28 and 60

62) limit 61 to yr="2003 - 2007"

63) practice guideline/

64) 28 and 63

65) limit 64 to yr="2003 - 2007"

**CRD**

1) [MeSH Low Back Pain EXPLODE 1 2 3](http://www.crd.york.ac.uk/CRDWeb/Search.aspx?SearchID=427854&SessionID=427854&D=98&E=96&H=76&SearchFor=MeSH Low Back Pain EXPLODE 1 2 3)

2) ["low back pain"](http://www.crd.york.ac.uk/CRDWeb/Search.aspx?SearchID=427855&SessionID=427854&D=154&E=101&H=89&SearchFor= "low back pain" )

3) ["lumbar pain"](http://www.crd.york.ac.uk/CRDWeb/Search.aspx?SearchID=427856&SessionID=427854&D=3&E=1&H=0&SearchFor= "lumbar pain" )

4) [lumbago](http://www.crd.york.ac.uk/CRDWeb/Search.aspx?SearchID=427857&SessionID=427854&D=4&E=1&H=0&SearchFor= lumbago )

5) [backache](http://www.crd.york.ac.uk/CRDWeb/Search.aspx?SearchID=427858&SessionID=427854&D=19&E=2&H=1&SearchFor= backache )

6) 1-5 OR

7) [MeSH Neck Pain EXPLODE 1 2 3](http://www.crd.york.ac.uk/CRDWeb/Search.aspx?SearchID=427860&SessionID=427854&D=28&E=9&H=5&SearchFor=MeSH Neck Pain EXPLODE 1 2 3)

8) [MeSH Whiplash Injuries EXPLODE 1](http://www.crd.york.ac.uk/CRDWeb/Search.aspx?SearchID=427861&SessionID=427854&D=10&E=3&H=4&SearchFor=MeSH Whiplash Injuries EXPLODE 1)

9) ["neck pain"](http://www.crd.york.ac.uk/CRDWeb/Search.aspx?SearchID=427862&SessionID=427854&D=52&E=12&H=14&SearchFor= "neck pain" )

10) ["mechanical neck disorder*"](http://www.crd.york.ac.uk/CRDWeb/Search.aspx?SearchID=427863&SessionID=427854&D=17&E=0&H=0&SearchFor= "mechanical neck disorder*" )

11) ["cervical pain*"](http://www.crd.york.ac.uk/CRDWeb/Search.aspx?SearchID=427865&SessionID=427854&D=2&E=1&H=2&SearchFor= "cervical pain*" )

12) [whiplash](http://www.crd.york.ac.uk/CRDWeb/Search.aspx?SearchID=427866&SessionID=427854&D=24&E=3&H=7&SearchFor= whiplash )

13) 7-12 OR

14) [MeSH Shoulder Pain EXPLODE 1 2 3](http://www.crd.york.ac.uk/CRDWeb/Search.aspx?SearchID=427868&SessionID=427854&D=9&E=5&H=1&SearchFor=MeSH Shoulder Pain EXPLODE 1 2 3)

15) [MeSH Rotator Cuff EXPLODE 1 2](http://www.crd.york.ac.uk/CRDWeb/Search.aspx?SearchID=427869&SessionID=427854&D=5&E=5&H=5&SearchFor=MeSH Rotator Cuff EXPLODE 1 2)

16) [MeSH Bursitis EXPLODE 1](http://www.crd.york.ac.uk/CRDWeb/Search.aspx?SearchID=427870&SessionID=427854&D=1&E=0&H=1&SearchFor=MeSH Bursitis EXPLODE 1)

17) ["shoulder pain"](http://www.crd.york.ac.uk/CRDWeb/Search.aspx?SearchID=427872&SessionID=427854&D=39&E=8&H=5&SearchFor= "shoulder pain" )

18) ["rotator cuff disorder*"](http://www.crd.york.ac.uk/CRDWeb/Search.aspx?SearchID=427873&SessionID=427854&D=1&E=0&H=0&SearchFor= "rotator cuff disorder*" )

19) ["frozen shoulder"](http://www.crd.york.ac.uk/CRDWeb/Search.aspx?SearchID=427874&SessionID=427854&D=3&E=0&H=0&SearchFor= "frozen shoulder" )

20) ["adhesive capsulitis"](http://www.crd.york.ac.uk/CRDWeb/Search.aspx?SearchID=427876&SessionID=427854&D=6&E=0&H=0&SearchFor= "adhesive capsulitis" )

21) 14-20 OR

22) 6 OR 13 or 21

23) [23 RESTRICT YR 2003 2007](http://www.crd.york.ac.uk/CRDWeb/Search.aspx?SearchID=427882&SessionID=427854&D=140&E=65&H=60&SearchFor=#23%20RESTRICT%20YR%202003%202007)

**Cochrane**

[1](http://212.169.42.7/newgenClibPlus/ASP/History.asp?updhist=1#%23)) [LOW BACK PAIN expandir todos los árboles (MeSH)](http://212.169.42.7/newgenClibPlus/ASP/srchResults.asp?histNo=1)

[2](http://212.169.42.7/newgenClibPlus/ASP/History.asp?updhist=1#%23)) [(low:ti next back:ti next pain:ti)](http://212.169.42.7/newgenClibPlus/ASP/srchResults.asp?histNo=2)

[3](http://212.169.42.7/newgenClibPlus/ASP/History.asp?updhist=1#%23)) [(low:ab next back:ab next pain:ab)](http://212.169.42.7/newgenClibPlus/ASP/srchResults.asp?histNo=3)

[4](http://212.169.42.7/newgenClibPlus/ASP/History.asp?updhist=1#%23)) [(lumbar next pain)](http://212.169.42.7/newgenClibPlus/ASP/srchResults.asp?histNo=4)

[5](http://212.169.42.7/newgenClibPlus/ASP/History.asp?updhist=1#%23)) [lumbago](http://212.169.42.7/newgenClibPlus/ASP/srchResults.asp?histNo=5)

[6](http://212.169.42.7/newgenClibPlus/ASP/History.asp?updhist=1#%23)) [backache](http://212.169.42.7/newgenClibPlus/ASP/srchResults.asp?histNo=6)

7) [(complaint* near back)](http://212.169.42.7/newgenClibPlus/ASP/srchResults.asp?histNo=24)

[8](http://212.169.42.7/newgenClibPlus/ASP/History.asp?updhist=1#%23)) [NECK PAIN expandir todos los árboles (MeSH)](http://212.169.42.7/newgenClibPlus/ASP/srchResults.asp?histNo=7)

[9](http://212.169.42.7/newgenClibPlus/ASP/History.asp?updhist=1#%23)) [WHIPLASH INJURIES expandir todos los árboles (MeSH)](http://212.169.42.7/newgenClibPlus/ASP/srchResults.asp?histNo=8)

10) [(neck:ti next pain:ti)](http://212.169.42.7/newgenClibPlus/ASP/srchResults.asp?histNo=9)

[1](http://212.169.42.7/newgenClibPlus/ASP/History.asp?updhist=1#%23)1) [(neck:ab next pain:ab)](http://212.169.42.7/newgenClibPlus/ASP/srchResults.asp?histNo=10)

[1](http://212.169.42.7/newgenClibPlus/ASP/History.asp?updhist=1#%23)2) [(mechanical next neck next disorder*)](http://212.169.42.7/newgenClibPlus/ASP/srchResults.asp?histNo=11)

[1](http://212.169.42.7/newgenClibPlus/ASP/History.asp?updhist=1#%23)3) [(cervical next pain)](http://212.169.42.7/newgenClibPlus/ASP/srchResults.asp?histNo=12)

[1](http://212.169.42.7/newgenClibPlus/ASP/History.asp?updhist=1#%23)4) [whiplash](http://212.169.42.7/newgenClibPlus/ASP/srchResults.asp?histNo=13)

15) [(complaint* near neck)](http://212.169.42.7/newgenClibPlus/ASP/srchResults.asp?histNo=23)

[1](http://212.169.42.7/newgenClibPlus/ASP/History.asp?updhist=1#%23)6) [SHOULDER PAIN expandir todos los árboles (MeSH)](http://212.169.42.7/newgenClibPlus/ASP/srchResults.asp?histNo=14)

[1](http://212.169.42.7/newgenClibPlus/ASP/History.asp?updhist=1#%23)7) [ROTATOR CUFF expandir todos los árboles (MeSH)](http://212.169.42.7/newgenClibPlus/ASP/srchResults.asp?histNo=15)

[1](http://212.169.42.7/newgenClibPlus/ASP/History.asp?updhist=1#%23)8) [BURSITIS expandir todos los árboles (MeSH)](http://212.169.42.7/newgenClibPlus/ASP/srchResults.asp?histNo=16)

[19](http://212.169.42.7/newgenClibPlus/ASP/History.asp?updhist=1#%23)) [(shoulder:ti next pain:ti)](http://212.169.42.7/newgenClibPlus/ASP/srchResults.asp?histNo=17)

[20](http://212.169.42.7/newgenClibPlus/ASP/History.asp?updhist=1#%23)) [(shoulder:ab next pain:ab)](http://212.169.42.7/newgenClibPlus/ASP/srchResults.asp?histNo=18)

[21](http://212.169.42.7/newgenClibPlus/ASP/History.asp?updhist=1#%23)) [(rotator next cuff next disorder*)](http://212.169.42.7/newgenClibPlus/ASP/srchResults.asp?histNo=19)

22) [(frozen next shoulder)](http://212.169.42.7/newgenClibPlus/ASP/srchResults.asp?histNo=20)

[23](http://212.169.42.7/newgenClibPlus/ASP/History.asp?updhist=1#%23)) [(adhesive next capsulitis)](http://212.169.42.7/newgenClibPlus/ASP/srchResults.asp?histNo=21)

[2](http://212.169.42.7/newgenClibPlus/ASP/History.asp?updhist=1#%23)4) [(complaint* near shoulder)](http://212.169.42.7/newgenClibPlus/ASP/srchResults.asp?histNo=22)

[25](http://212.169.42.7/newgenClibPlus/ASP/History.asp?updhist=1#%23)) 1-7 OR

[26](http://212.169.42.7/newgenClibPlus/ASP/History.asp?updhist=1#%23)) [8-15](http://212.169.42.7/newgenClibPlus/ASP/srchResults.asp?histNo=26) OR

[27](http://212.169.42.7/newgenClibPlus/ASP/History.asp?updhist=1#%23)) 16-24 OR

[28](http://212.169.42.7/newgenClibPlus/ASP/History.asp?updhist=1#%23)) [25-27](http://212.169.42.7/newgenClibPlus/ASP/srchResults.asp?histNo=28) OR

[29](http://212.169.42.7/newgenClibPlus/ASP/History.asp?updhist=1#%23)) [28 (2003 to present)](http://212.169.42.7/newgenClibPlus/ASP/srchResults.asp?histNo=29)

**TRIP DATABASE**

1) whiplash or (neck and pain) or (cervical and pain) 🡪 Title

2) (shoulder and pain) or (rotator and cuff) or (frozen and shoulder) or (adhesive and capsulitis) 🡪 Title

3) (low back pain) or lumbago or (lumbar and pain) or backache

**PUBGLE**

1) (low back pain) or lumbago or (lumbar and pain) or backache

2) (shoulder and pain) or (rotator and cuff) or (frozen and shoulder) or (adhesive and capsulitis)

3) whiplash or (neck and pain) or (cervical and pain)

**National Guideline Clearinghouse**

Whiplash

“Neck pain”

“Cervical pain”

“rotator cuff”

“frozen shoulder”

“adhesive capsulitis”

“low back pain”

“lumbar pain”

**Fisterra**

Clinical Guidelines 🡪 Pain

**GuíaSalud**

Catalog of Clinical Practice Guidelines for the Spanish NHS. Selection by Index: Osteomuscular and Connective Tissue Diseases.

**Web de la espalda**

Evidence Based Clinical Practice Guidelines

**European Commission Research Directorate General**

Working Group 1: Acute low back pain 🡪 Results (guidelines)

Working Group 2: Chronic low back pain 🡪 Results (guidelines)

**ICSI. Institute for Clinical Systems Improvement**

Guidelines & more: Selection by Index of: Musculo-skeletal disorders
